# 3D Copper Foam-Supported CuCo_2_O_4_ Nanosheet Arrays as Electrode for Enhanced Non-Enzymatic Glucose Sensing

**DOI:** 10.3390/s18041131

**Published:** 2018-04-08

**Authors:** Fangqing Liu, Yi Zhuang, Mingliang Guo, Yongjun Chen, Jinchun Tu, Lei Ding

**Affiliations:** State Key Laboratory of Marine Resource Utilization in South China Sea, College of Materials and Chemical Engineering, Hainan University, Haikou 570228, China; lfqhndx2015@163.com (F.L.); zy839091348@aliyun.com (Y.Z.); guomingliang02@163.com (M.G.); yongchen@hainu.edu.cn (Y.C.); tujinchun@hainu.edu.cn (J.T.)

**Keywords:** glucose, sensors, Cu foam, CuCo_2_O_4_

## Abstract

CuCo_2_O_4_ anchored on Cu foam (CuCo_2_O_4_/CF) with polycrystalline features was fabricated by a mild process based on solvothermal reaction and subsequent calcination in this work. The structure and morphology of the obtained materials were thoroughly characterized by X-ray diffraction, X-ray photoelectron spectroscopy, field-emission scanning electron microscopy, and transmission electron microscopy. According to the above analysis, the morphology of the CuCo_2_O_4_ was nanosheet arrays. Meanwhile, the CuCo_2_O_4_ was grown on Cu foam successfully. The CuCo_2_O_4_/CF displayed good electrochemical properties for glucose detection at a linear range from 0 mM to 1.0 mM. Meanwhile, the detection limit was as low as 1 μM (S/N = 3), and the sensitivity was 20,981 μA·mM^−1^·cm^−2^. Moreover, the selectivity and the stability were tested with excellent results. This nanomaterial could show great potential application in electrochemical sensors.

## 1. Introduction

High-precision glucose detection is a growing demand for social development. It has been widely used in various fields such as biotechnology, food factories, and ecological environments [1]. Currently, many strategies are available to monitor glucose. Electrochemical sensor can transform the signals of chemical reaction on the electrode surface into electrical signals. These signals can be easily recorded and quantified. Therefore, this method is suitable for the rapid and precise monitoring of glucose [2]. In general, electrochemical sensor could be classified into electrochemical enzymatic sensor and electrochemical non-enzymatic sensor. To date, many studies reported that glucose oxidase-modified electrodes exhibit good sensitivity and high selectivity for glucose detection [3]. However, the development of electrochemical enzymatic sensor is limited because of the high price of the active enzyme. Moreover, the pH and temperature in the environment could easily exhibit a negative impact on the performance during detection. Thus, the research on non-enzymatic glucose sensors, which could reveal high stability, has drawn great attention.

CuCo_2_O_4_ has attracted considerable attention in recent research on non-enzymatic glucose sensors. CuCo_2_O_4_ has higher electrical conductivity and electrochemical activity than simple metal oxides because of a relatively low activation energy for electronic transmission between multiple transition-metal cations [4]. Consequently, the variety nanostructure of CuCo_2_O_4_, such as nanoflakes [5], nanowires [6], and nanoparticles [7], has been widely applied in Li-ion batteries [8], supercapacitors [4], and sensors [9]. In a recent study, hollow CuCo_2_O_4_-functionalized porous graphene composite was prepared successfully by Zhao et al. They used this composite as electrode materials to detect glucose with high sensitivity of 2426 μA·mM^−1^·cm^−2^ [10]. The performance of glucose sensors also significantly relied on the electrochemical properties of the electrodes in addition to the appropriate immediate constituents of active materials. In previous research, active nanomaterials directly growing on conductive substrates without polymer binders have become a great prospect because the “dead surface” from the direct contact with the electrolyte taking part in the Faradaic reactions could be decreased effectively [11]. Reliably, some works [12,13] demonstrated a remarkably improved detection sensitivity when the utilization of CuCo_2_O_4_ nanosheets were grown on graphite paper and nanowires on carbon cloths in non-enzymatic glucose sensor. To fabricate a notable breakthrough, the electrochemical performance still needs to be further improved. Notably, copper foam is a popular material with three-dimensional network structure. Copper foam shows the advantages of high conductivity, cheap price, and convenient synthesis [14]. It can also be used as a conductive substrate to supply active sites, and the electrode preparation can be simplified. Herein, the copper foam is a potential material, which can be applied in the research of glucose sensor.

In this report, CuCo_2_O_4_ nanosheet arrays on a copper foam substrate were synthesized by a simple solvothermal reaction and subsequent calcination. Then, they were used as electrode to detect glucose. Some of the drawbacks caused by the use of a binder were avoided because the copper foam-conductive substrate acted directly as an electrode. Moreover, the three-dimensional network of copper foam provided a large surface area for the loading of the material, resulting in several active site exposure on the electrode surfaces. Thus, the electrode material exhibited an excellent performance in glucose detection.

## 2. Materials and Methods

### 2.1. Chemicals

Cu foam was obtained from Jiangxi Chemical Reagent Factory (Nanchang, China). Co(NO_3_)_2_, Cu(NO_3_)_2_, hydrochloric acid, and NaOH were purchased from the Sinopharm Chemical Reagent Co., Ltd. (Shanghai, China). Ascorbic acid (AA), urea, uric acid (UA), and dopamine (DA) were bought from Guangzhou Chemical Reagent Factory (Guangzhou, China). Isopropanol and absolute ethanol were purchased from Guangdong Guanghua Sci.-Tech. Co. (Guangzhou, China). All chemical reagents used in the experiment were of analytical grade and without any further purification. All the aqueous solutions were prepared in deionized water.

### 2.2. Fabrication of CuCo_2_O_4_ Nanosheets

Cu foam (CF) was cut into 2 cm × 3 cm slice and cleaned with the help of sonication in acetone, water, and 3.0 M HCl in sequence. Then, the cleaned Cu foam was washed quickly with ultrapure water and dried with pure nitrogen gas. The CuCo_2_O_4_ nanosheet arrays on Cu foam substrate were fabricated via a hydrothermal reaction. In a typical process, 18.475 mg of Cu(NO_3_)_2_·6H_2_O and 36.375 mg of Co(NO_3_)_2_·6H_2_O were added into 40 mL isopropanol under magnetic stirring. In this case, a homogeneous apparent solution could be obtained. Subsequently, the solution was transferred into a Teflon-lined stainless steel autoclave, and the cleaned Cu foam was immersed into the solution. The autoclave was sealed and kept at 120 °C for 12 h in an electric oven. Finally, it was cooled naturally at room temperature. The Cu foam with precursor was removed from the autoclave. Then, it was washed with deionized water and absolute alcohol thoroughly before drying in air. Finally, the obtained samples were annealed at 350 °C for 2 h.

### 2.3. Characterizations

The crystallographic structure of samples was characterized by X-ray diffraction (XRD, D8-Discovery Bruker, Karlsruhe, Germany, 40 kV, 30 mA, Cu Kα, λ = 1.5406 Å). The morphology of the as-obtained products was recorded using scanning electron microscopy (SEM, Hitachi S-3000 4800, Tokyo, Japan) and transmission electron microscopy (TEM, JEOL-2100F microscope, Tokyo, Japan). X-ray photoelectron spectroscopy (XPS, TENSOR27, Karlsruhe, Germany) was tested to make a thorough inquiry about the chemical bonding status of the CuCo_2_O_4_ materials.

### 2.4. Electrochemical Measurements

All the electrochemical measurements were performed with a typical three-electrode system by using SP-200 Electrochemical Workstation (Bio-Logic Science Instruments, Paris, France). The CuCo_2_O_4_/CF was cut into a small pieces and directly served as the working electrode, and a platinum wire and a saturated Ag/AgCl electrode was utilized as counter and reference electrodes, respectively. All electrochemical measurements were tested in 0.1 M NaOH solution at room temperature (25 °C). The amperometric response tests were measured at an appropriate potential under stirring condition, and the analytes were added into the electrolyte after the background currents decay to a steady-state.

## 3. Results and Discussion

**Figure 1:**
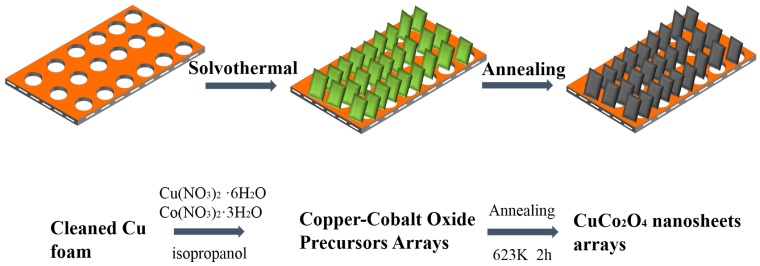
Illustration fabrication route of CuCo_2_O_4_ nanosheets on Cu foam.

Figure 1 schematically illustrates the synthesis of CuCo_2_O_4_ nanosheets on a foam copper as electrode materials. First, the copper foam was ultrasonically cleaned in water, alcohol, and 3.0 M HCl successively to remove some of the surface impurities. Then, CuCo_2_O_4_ nanosheet arrays were grown on a Cu foam substrate via a hydrothermal and calcination process. The Cu foam could be directly served as electrode because of its good electrical conductivity and lightweight.

**Figure 2:**
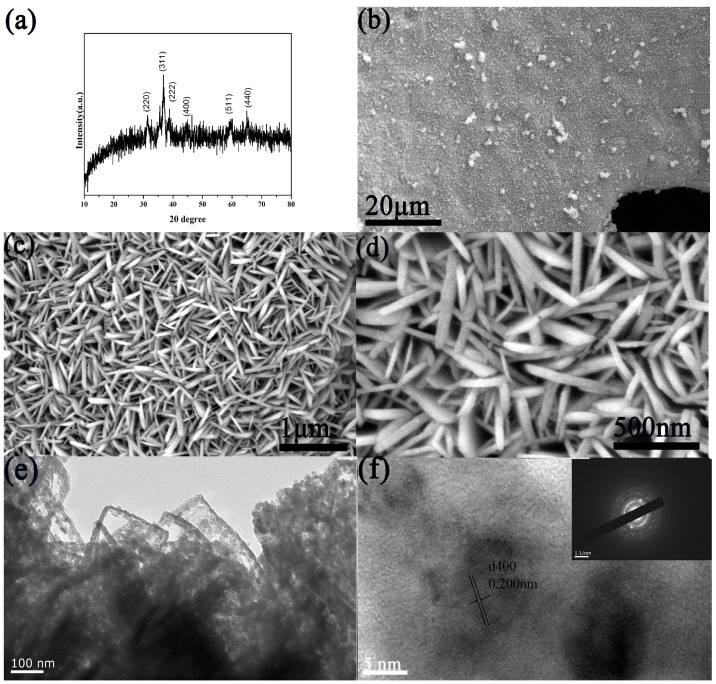
(**a**) XRD patterns and (**b**–**d**) the FESEM images of the CuCo_2_O_4_ nanosheets; (**e**) TEM and (**f**) HRTEM images of the CuCo_2_O_4_ nanosheets. The inset of (**f**) shows the SAED pattern.

Figure 2a shows the XRD of the samples. As seen in Figure 2a, the well-defined diffraction peaks could be indexed to the cubic spinel CuCo_2_O_4_ (JCPDS Card NO.01-1155), the diffraction peaks positioned at 31.0°, 36.6°, 38.3°, 44.5°, 58.9°, 64.8° can be identified as (220), (311), (222), (400), (511), (400). Confirming that the CuCo_2_O_4_ was successfully prepared on a copper foam. The structure was investigated by the field-emission SEM (FESEM). The surface of 3D Cu foam was deposited fully and uniformly, indicating that the CuCo_2_O_4_ was grown well on the substrate as shown in Figure 2b. The FESEM in Figure 2c,d further revealed the morphology of CuCo_2_O_4_. Figure 2c,d display that the CuCo_2_O_4_ morphology was nanosheets, and its thickness was approximately 50 nm. Thus, the thin nanosheets could provide several active sites for glucose oxidation. The TEM imaging was performed to further investigate the structure of CuCo_2_O_4_ nanosheets. As shown in Figure 2e, some porous structures were found in the nanosheets, possibly caused by the release of gas or water molecules during the annealing treatment [15]. Figure 2f shows well-resolved lattice fringes with interplanar distances of 0.20 nm, corresponding to the (400) planes of CuCo_2_O_4_. The selected-area electron diffraction (SAED) pattern in the inset (Figure 2f) demonstrated the polycrystalline structure of the CuCo_2_O_4_ nanosheets.

**Figure 3:**
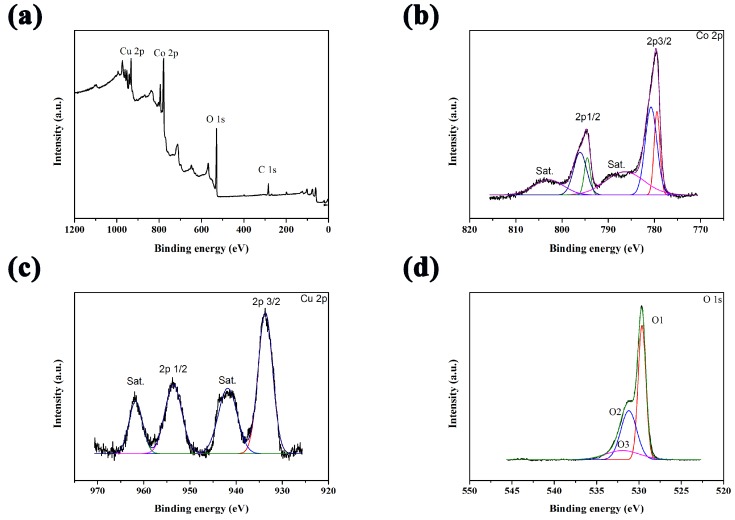
(**a**) XPS survey spectrum, XPS spectra of (**b**) Co 2p, (**c**) Cu 2p, and (**d**) O 1s for CuCo_2_O_4_ nanosheets.

Additional data were obtained by XPS measurements to further determine the surface characteristics of these materials. The survey spectrum (Figure 3a) showed the presence of Cu, Co, O, and C. In the Co 2p spectrum (Figure 3b), two main peaks located at binding energies of 780.0 and 794.4 eV could be assigned to Co 2p_3/2_ and Co 2p_1/2_, respectively, indicating the existence of both Co^3+^ and Co^2+^ [16]. Figure 3c shows the Cu 2p spectrum. Four peaks were observed at binding energies of 934.4, 942.7, 954.2, and 962.0 eV. The main peaks located at 934.4 and 954.2 eV could be assigned to Cu 2p_3/2_ and Cu 2p_1/2_, and the shakeup satellite peaks located at 942.8 and 962.0 eV could confirm the characteristic of the Cu^2+^ oxidation state [17]. Three components (O1, O2, and O3) were present in the O 1s spectra (Figure 3d) 529.6, 531.4, and 532.4 eV, corresponding to the metal–oxygen bonds, large numbers of defect, and the multicity of physisorbed and chemisorbed water into and near the surface, respectively [18]. The XPS results above coincided with XRD results, confirming the successful formation of CuCo_2_O_4_ phase.

**Figure 4:**
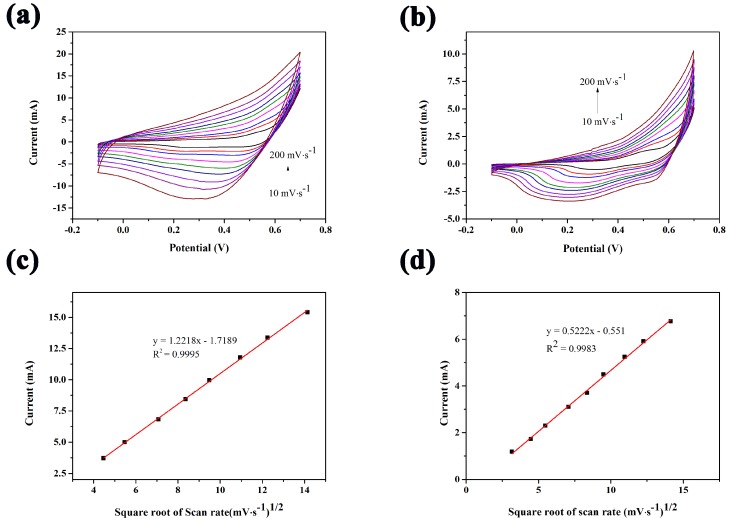
CV curves of (**a**) CuCo_2_O_4_ nanosheets on Cu foam and (**b**) bare Cu foam electrodes measured in a 0.1 M NaOH at various scan rates (10–200 mV·s^−1^). (**c**,**d**) Corresponding plots of current vs. the square root of scan rate for CuCo_2_O_4_/Cu foam and bare Cu foam, respectively.

Cyclic voltammetry (CV) of the CuCo_2_O_4_/CF electrode was tested in the presence of 0.1 mol/L NaOH solution at different scan rates from 10 mV/s to 200 mV/s. Both the anodic and cathodic peak current densities increased with the increase of scan rate varying from 10 mV/s to 200 mV/s as shown in Figure 4a. Figure 4c shows the corresponding linear curves, indicating a good linear relationship with a correlation coefficient of 0.9995. This phenomenon indicated that the electrochemical reaction on the CuCo_2_O_4_/CF electrode was a diffusion-controlled process.

The electroactive surface area (A) was estimated to demonstrate the effective surface area of the CuCo_2_O_4_/CF electrode by using the Randles–Sevcik Equation [19] as follows:

*I_p_* =2.69 × 10^5^ × n^3/2^AD^1/*2*^*v^1^*^/2^C (1)

where A is the effective surface area (cm^2^); *I_p_* is the peak current of the redox reaction; n is the number of electrons transferred; D is the diffusion coefficient; *v* is the scan rate (V·s^−1^), and C is the reactant concentration. Figure 4b,d show the CV curves of bare Cu foam electrode at different scan rates and fitted curve. The slope of the fitted curve was lesser than that of the CuCo_2_O_4_/CF electrode. This result implied that the electrochemical active surface area of CuCo_2_O_4_/CF electrode was higher than that of Cu foam electrode according to the Randles–Sevcik equation.

**Figure 5:**
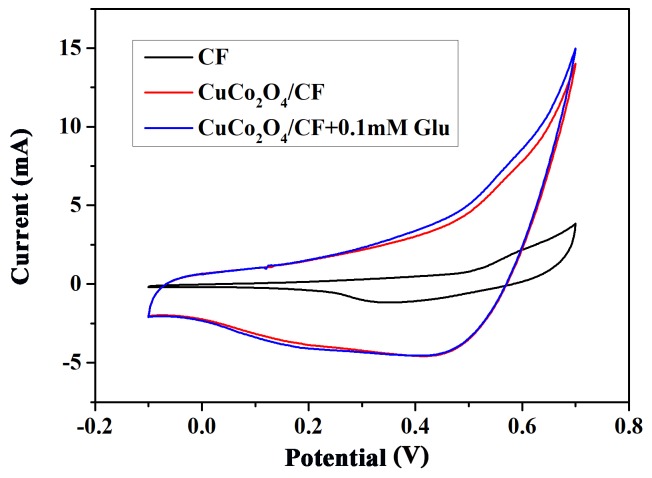
CV curves of bare Cu foam (black line) and CuCo_2_O_4_ nanosheets in the absence (red line) and presence (blue line) of 0.1 mM glucose in 0.1 M NaOH at 50 mV·s^−1^.

In this work, the prepared CuCo_2_O_4_/CF directly served as working electrode for glucose detection. The CV was recorded in 0.1 mol/L NaOH solution in the absence and presence of 0.1 mM glucose as shown in Figure 5. As a reference, the blank Cu foam electrode showed no obvious redox peak, implying a poor electrocatalytic activity. For the CuCo_2_O_4_/CF electrode, two sensitive oxidative peaks could be observed at 0.4 and 0.56 V in the CV curve. Similar to many other Cu-based glucose non-enzyme sensors, the oxidative current of Cu^2+^/Cu^3+^ positioned at approximately 0.4 V was not obvious [13]. Therefore, the oxidative peaks at 0.4 V could be ascribed to the reversible transition between Co_3_O_4_ and CoOOH, whereas the oxidative peaks at 0.56 V could be ascribed to the reversible transition between and transition between CoOOH and CoO_2_ [20]. The reversible reaction mechanisms of CuCo_2_O_4_ species in the alkaline electrolyte could be expressed in Equations (2) and (3) [21]. The introduction of 0.1 mM glucose caused an obvious increase in the anodic peak currents. This phenomenon could be ascribed to the glucose oxidation to gluconolactone, which was accompanied by the conversion of CoO_2_ to CoOOH and M(Cu, Co)OOH to M(OH)_2_. The possible electro-oxidation mechanism of glucose could be expressed in Equations (4)–(6) [22]. The observed result demonstrated that the CuCo_2_O_4_/CF exhibited a high-efficient catalysis of glucose electro-oxidation.

CuCo_2_O_4_ + OH^−^ + H_2_O → CuOOH + 2CoOOH + e^−^ (2)

CoOOH + OH^−^ → CoO_2_ + H_2_O + e^−^ (3)

2CoO + C_6_H_10_O_6_ (glucose) → 2CoOOH + C_6_H_10_O_6_ (gluconolactone) (4)

CoOOH + C_6_H_10_O_6_ (glucose) → Co(OH)_2_ + C_6_H_10_O_6_ (5)

CuOOH + C_6_H_10_O_6_ (glucose) → Cu(OH)_2_ + C_6_H_10_O_6_ (6)

**Figure 6:**
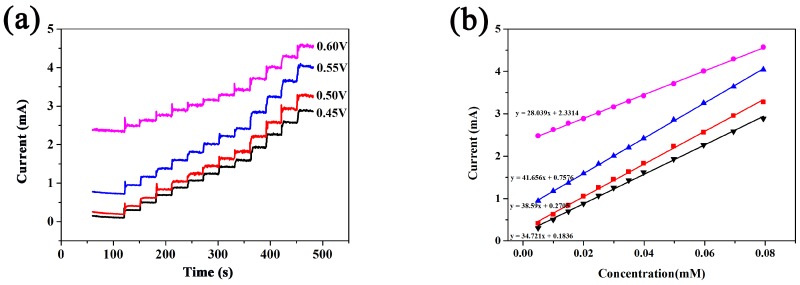
(**a**) Amperometric response with successive addition of 10 mM glucose at different potentials. (**b**) The corresponding calibration curve for glucose oxidation.

We selected a proper working potential for glucose determination. Figure 6a shows the amperometric response curves of CuCo_2_O_4_/CF to stepwise addition of 0.1 mM glucose into 0.1 M NaOH solution at different potentials between +0.45 V and +0.6 V. Figure 6b shows the corresponding Calibration curve. Obviously, the current response toward glucose improved with the increasing potential from 0.45 V to 0.55 V and then initially decreased from 0.55 V to 0.60 V, indicating that +0.55 V is the most appropriate potential for glucose sensing. Therefore, subsequent amperometric response to glucose was executed at the potential of +0.55 V.

**Figure 7:**
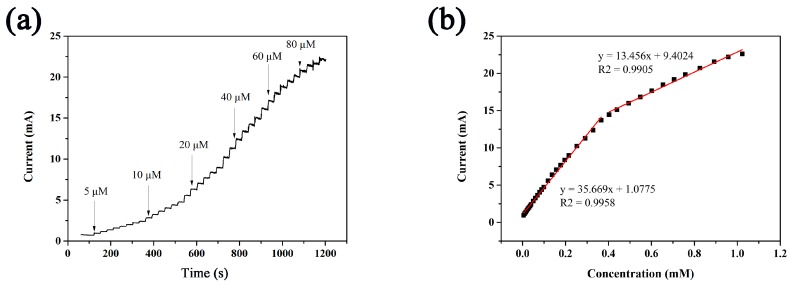
(**a**) Amperometric response of CuCo_2_O_4_/CF to the successive addition of the glucose solution with different concentrations in 0.1 M NaOH solution; (**b**) calibration curve for glucose oxidation on CuCo_2_O_4_/CF.

Figure 7a shows the amperometric responses of the CuCo_2_O_4_/CF electrode to successive addition of glucose at the potential of +0.55 V vs. Ag/AgCl. The current was improved rapidly with the addition of glucose, indicating its stable and fast-response performance for glucose detection. At a glucose concentration of between 0.4 and 1.1 mM, the current response decreased gradually possibly be due to the faster consumption than the glucose diffusion, the local pH change, or the oxidized intermediate adsorption on active sites [23]. Figure 7b shows the corresponding calibration curve for glucose sensors, demonstrating a linear range from 0 mmol/L to 0.4 mmol/L glucose with a sensitivity of 20,981 μA·cm^−2^·mM^−1^, a correlation coefficient of 0.9958, a linear concentration range from 0.4 mmol/L to 1.0 mmol/L glucose with a sensitivity of 7915 μA·cm^−2^·mM^−1^, and a correlation coefficient of 0.9905. On the basis of the S/N = 3, the limit of detection (LOD) was calculated to reach 1.0 μM. The results suggested that the electrochemical behavior of the CuCo_2_O_4_/CF nanomaterial exhibited low detection limit, high sensitivity, and quick response time on glucose detection. The performance of the CuCo_2_O_4_/CF electrode was compared with other previously reported non-enzymatic glucose sensor based on Cu, Co, and self-supporting substrate as shown in Table 1. The CuCo_2_O_4_/CF electrode showed a low LOD, wide linear range, and ultrahigh sensitivity. The high performance could be attributed to that CuCo_2_O_4_ possessed excellent electrochemical properties and high conductivity in the synergistic effect of copper and cobalt ions. Meanwhile, the morphology of ultrathin nanosheet arrays could improve the surface area and offer many catalytic sites for glucose oxidation. Moreover, 3D Cu foam as conductive substrate can provide several catalyst loadings and enhanced electron transport instead of the use of polymer binders [24].

**Figure 8:**
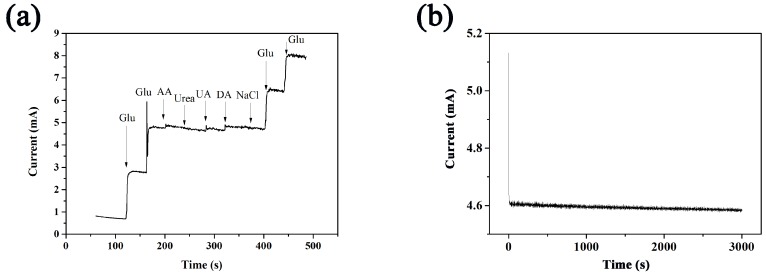
(**a**) Interference test of CuCo_2_O_4_ upon addition of successive addition of 50 μM glucose, 5 μM AA, 5 μM urea, 5 μM UA, 5 μM DA, 5 μM NaCl, and 5 μM glucose at the potential of 0.55 V in 0.1 M NaOH solution; (**b**) Time-current curves of CuCo_2_O_4_ at the potential of 0.55 V in 0.1 M NaOH solution.

Selectivity is another important analysis parameter for glucose sensors. Therefore, to carry out anti-interference analysis is essential. AA, urea, UA, and DA would interfere the measurement results because they showed the analogous electrocatalysis behavior to the oxidation of glucose and usually existed together with glucose in human serum samples. Thus, the anti-interference performance of CuCo_2_O_4_/CF toward these interfering species was studied by amperometric method. Glucose aqueous solution of 50 μmol/L was added into 0.1 mol/L NaOH aqueous solution, and 5 μmol/L AA, 5 μmol/L urea, 5 μmol/L UA, 5 μmol/L DA, and 0.1 mmol/L NaCl were continuously added into 0.1 mol/L NaOH solution under stirring. The amperometric reaction is observed in Figure 8a. The current response caused by the interference was weak and almost negligible, indicating that the electrode exhibited good selectivity in glucose sensors.

Finally, the stability of CuCo_2_O_4_/CF was examined. Stability was performed through testing their steady-state current response over a period of 3000 s in a 0.1 mol/L NaOH with 100 μM glucose solution. As shown in Figure 8b, at the end of 3000 s, the deviation of current response was merely 1.70%. The result within a reasonable margin of error revealed good stability of glucose sensing.

## 4. Conclusions

In summary, CuCo_2_O_4_ nanosheets grown on 3D Cu foam was successfully fabricated by solvothermal synthesis and subsequent calcined treatment. The structure and composition of materials were characterized by various instruments. CuCo_2_O_4_ as a binary metal oxide based on 3D Cu foam without binder was beneficial for mass electron transport and electrochemical oxidation of glucose. The electrochemical behavior was performed with high sensitivity (20,981 μA·cm^−2^ mM^−1^), low detection limit (1 μmol/L), and quick response times. In addition, the remarkable anti-interference made it possible in practical application. The superior glucose sensing properties should be attributed to the following reasons: The spinel structure of CuCo_2_O_4_ conducive to enhance the intrinsic catalytic activity and electronic transfer property, the morphology of nanosheet increase the specific surface area and provide more active sites, the in-situ growing hierarchical CuCo_2_O_4_ on 3D Cu foam helps to reduce the contact potential on the electrode and increase the electron collecting properties Furthermore, with the advantages of high sensitivity, good selectivity, and low cost, Cu foam was a great substrate material. Thus, Cu foam might be potential in other electrochemical studies.

## Figures and Tables

**Table 1:** Performance comparison of CuCo_2_O_4_/CF with previous reports.

Electrode Material	Linear Range (mM)	Detection Limit (μM)	Sensitivity (μA·mM^−1^·cm^−2^)	Reference
CuCo_2_O_4_ nanosheet on graphite paper	Up to 0.32	0.55	3625	[12]
CuCo_2_O_4_ nanosheet on ITO	0.005–0.11	5.2	8250	[25]
CuCo_2_O_4_ NWAs/CC	0.001–0.93	0.5	3930	[13]
CuO/Cu nanomaterials	Up to 4	0.5	4201	[26]
CoOOH nanosheet array	0.003–1.109	1.37	526	[27]
Cu_2_O/Cu foam	0.001–1.4	0.13	5040	[28]
CuO/Ni foam	0.0005–3.5	0.16	1084	[29]
CuCo_2_O_4_/Cu foam	Up to 1.0	1	20,981	This work
